# 3D bioprinting and Rigenera^®^ micrografting technology: A possible countermeasure for wound healing in spaceflight

**DOI:** 10.3389/fbioe.2022.937709

**Published:** 2022-08-30

**Authors:** Flaminia Aliberti, Elisa Paolin, Laura Benedetti, Gabriella Cusella, Gabriele Ceccarelli

**Affiliations:** ^1^ Department of Public Health, Experimental and Forensic Medicine, Human Anatomy Unit, University of Pavia, Pavia, Italy; ^2^ Fondazione IRCCS Policlinico San Matteo, Center for Inherited Cardiovascular Diseases, Transplant Research Area, Pavia, Italy; ^3^ Human Brain Wave, Turin, Italy

**Keywords:** microgravity, 3D bioprinting, micrografting, skin, regenerative medicine, wound healing, space, tissue engineering

## Abstract

Plant and animal life forms have progressively developed mechanisms for perceiving and responding to gravity on Earth, where homeostatic mechanisms require feedback. Lack of gravity, as in the International Space Station (ISS), induces acute intra-generational changes in the quality of life. These include reduced bone calcium levels and muscle tone, provoking skin deterioration. All these problems reduce the work efficiency and quality of life of humans not only during exposure to microgravity (µG) but also after returning to Earth. This article discusses forthcoming experiments required under gravity and µG conditions to ensure effective and successful medical treatments for astronauts during long-term space missions, where healthcare is difficult and not guaranteed.

## 1 Introduction

Wound healing (WH) is a dynamic and complex biological process consisting of tightly coordinated interactions between growth factors, cytokines, chemokines, different cell types, extracellular matrix (ECM), and proteases (Nourian Dehkordi et al., 2019; Morbidelli et al., 2021). WH is conventionally separated into several events: coagulation, inflammation, granulation tissue formation, proliferation, and remodeling (Jimi et al., 2017; Cialdai et al., 2020). Alterations or blocking of one or more stages of the repair process can lead to the formation of chronic or intractable wounds, issues that may arise in astronauts during long-term space explorations (Riwaldt et al., 2021). Indeed, astronauts’ complaint of skin deterioration during space missions has been reported by the National Aeronautics and Space Administration (NASA) (Riwaldt et al., 2021; Garcia, 2022). Long-term exposure to µG induces mechanical stress upon mammalian tissue, leading to rapid alterations that increase the risk of physiological degeneration of the bone, muscles, cardiovascular capacity, and WH. High fibrinogen concentrations, the presence of thrombin, and endothelial damage markers were detected in the blood of astronauts in spaceflight missions in addition to the reduction of blood flow (Kim et al., 2021). Prolonged exposure to µG may also result in the development of anemia and the alteration of cardiac physiology (Trudel et al., 2020).

As the interest in space travel and identifying other habitable planets increases, it becomes more important to deepen our understanding of how a low-gravity environment may impair and affect the human body. In particular, the impacts of the absence of gravity, the absence of atmosphere, and the lack of Earth’s magnetic field on organ systems are under-researched.

Initial research by Blaber et al. (2014) reported experiments conducted in collaboration between NASA and the Russian Space Agency over the course of the Foton M2 and M3 missions, where various tests on tissue regeneration were conducted. Specifically, it was observed that µG is responsible for the inhibition of the transition from progenitor cells to differentiated adult cells, suggesting that prolonged exposure to µG may result in a decrease in the tissue regeneration capacity.

Furthermore, NASA designed an automated Bioculture System meant for use on the International Space Station (ISS) to carry out bioscience research exploiting ten independent cell culture cassettes, each with a standalone cell culture bioreactor to allow multiple experiments simultaneously (Blaber et al., 2014).

Among several approaches which have been developed to fully characterize the biological skin model, tissue engineering (TE) acquired a pivotal role due to its capability to produce three-dimensional (3D) biological *in vitro* models that mimic the skin’s physiological environment (Singer and Boyce, 2017). Indeed, Zhou et al. (2020) have designed and fabricated a functional living skin formed by the human skin fibroblast and biomimetic bioink, using the 3D bioprinting technology, which is one of the most innovative and promising techniques of tissue engineering. They obtained a 3D structure model capable of widely promoting cell viability, migration, and proliferation compared to 2D *in vitro* models. Furthermore, the *in vivo* application of the investigated model demonstrated very high performance in tissue regeneration and, above all, an acceleration in wound repair.

Moreover, another main goal of TE is to recreate biological *in vitro* substitutes with the aim of providing tissue models to be used in the replacement or regeneration of damaged tissues (Berthiaume et al., 2011). In this regard, Cao et al. (2020) have engineered a scaffold based on human-like collagen and carboxymethylated chitosan, whose geometry and composition allow to mimic the extracellular matrix intrinsic characteristics (transportation of materials, cell adhesion, and proliferation), making it an advantageous candidate as a project strategy for skin defect repair as it has been demonstrated by promising *in vivo* collected data. Hence, in the last decades, new materials, methodologies, and technologies have emerged for the creation of skin substitutes, including the use of arising methodologies (Tresoldi et al., 2019; Cubo-Mateo and Gelinsky, 2021; Tottoli et al., 2022). Considering the challenges that WH research encounters under normal gravity conditions, it is ambitious to identify a process to address WH under µG conditions. However, on board the ISS, it is necessary to consider the additional limitations that a medical treatment on board or an *in vitro* experiment would encounter, e.g., the number of devices and limited transportable materials, time constraints, changes in mass, chemical–physical properties, and the levels of containment to protect the crew, ferry vehicle, and ISS itself (Ferranti et al., 2021). The aim of this work is to discuss the forthcoming experiments that need to be carried out to study and characterize potential countermeasures to guarantee new, effective, and successful medical treatments for astronauts.

Rigenera^®^ (Human Brain Wave, Turin, Italy) is a new autologous micrografting technology (AMG), already successfully employed in WH (Uehara and Shimizu, 2019). It is also under investigation to assess its potential to favorably influence WH in a low-gravity environment. Furthermore, AMG technology, together with 3D bioprinting, is acquiring a pivotal role in the field of tissue engineering, removing limitations associated with autograft transplants (e.g., risk of infections, secondary diseases, and low compliance for the patient).

## 2 Biological design of a novel approach for wound healing in space

The skin is the largest single organ of the body. It is the main barrier to the external environment and, therefore, has a protective function (Gollnick et al., 2019).

Aging can lead to the development of skin fragility due to the defective migration of WH cells (Afshinnekoo et al., 2020). In space flights, environmental factors such as µG and radiation can have a severe impact on the skin, resulting in impaired WH mechanisms in case serious traumatic injuries occur (Tronnier et al., 2008; Braun et al., 2018; Cubo-Mateo and Gelinsky, 2021).

Among several re-epithelialization technologies developed to establish a physiological WH process, it has been demonstrated that the AMG technology is able to respond to the principal limitations to the current gold standard approach of autologous grafting. This includes the need to use large quantities of tissues, long sample preparation time, and long-term hospitalization (Niimi et al., 2022). It has been shown by Balli et al. (2020) that the Rigenera^®^ technology plays a key role in the re-epithelialization stage by modulating the genes responsible for angiogenesis, cell migration, and MAPK/ERK activation, and inducing the migration of fibroblasts. They observed quicker wound closure in cell scratch assays in the presence of AMGs. These findings could be tested under simulated µG conditions on Earth using a random positioning machine (RPM) to rapidly establish if AMGs could be employed as a potential solution for in-space medical care to investigate further in conjunction with a tissue-engineered scaffold (Cialdai et al., 2020). Subsequently, a gene expression analysis must be conducted, like RNAseq or microRNA analysis, to comprehensively evaluate the AMG technology.

Balli et al. (2020) suggested the investigation of all genes related to the matrix metalloproteinases (MMPs) and whose expressions increase during AMG treatment, leading to an expansion of chemokine regulators, remodeling due to myofibroblasts, and a faster wound contraction. Moreover, in the study by Cialdai et al. (2020), it was observed that when α-smooth muscle actin (α-SMA—gene related with fibroblast–myofibroblast transdifferentiation) expression decreased on fibroblasts exposed to µG, vascular endothelial growth factor (VEGF—gene active in the processes of angiogenesis and vasculogenesis and promotes cell migration) expression significantly increased.

Moving toward a higher level of complexity, along with the use of a solution of micrografts, it is also useful to exploit dermal substitutes designed to mimic the physiological architecture of the skin. For full-thickness wounds, the use of scaffolds would not only be useful but would also be necessary to provide a 3D structure to the solution of micrografts and ensure, therefore, a faster and more efficient WH (Negut et al., 2020).

The production of a suitable skin substitute has been a long-sought goal for modern medicine; in fact, in the last decades, significant advances have been made in the field of skin tissue engineering, both in the development of engineered substitutes useful for the replacement of skin lost due to trauma, wounds, or burns and as a realistic model to be used for *in vitro* tests. The design implementation of skin tissue is realized by combining materials, cells, biochemical mediators, and innovative culture systems (Berthiaume et al., 2011).

Pellegrini et al. developed a new life-saving therapeutic strategy to replace and repair severely damaged tissues using new substrates and new culture technologies. After isolation and characterization of keratinocytes (holoclones, meroclones, and paraclones), a fibrin scaffold was designed, and cells were seeded. The results show that cell percentage is maintained when cultured on fibrin, demonstrating that the fibrin scaffold allows the maintenance of the epidermal phenotype.

Moreover, *in vivo* experiments have been carried out using the bioconstruct (scaffold and seeded keratinocytes), which has been shown to support permanent cell proliferation and ensure high reproducibility if it is applied to injured skin regions (Graziella and Luca, 1999).

**FIGURE 1:**
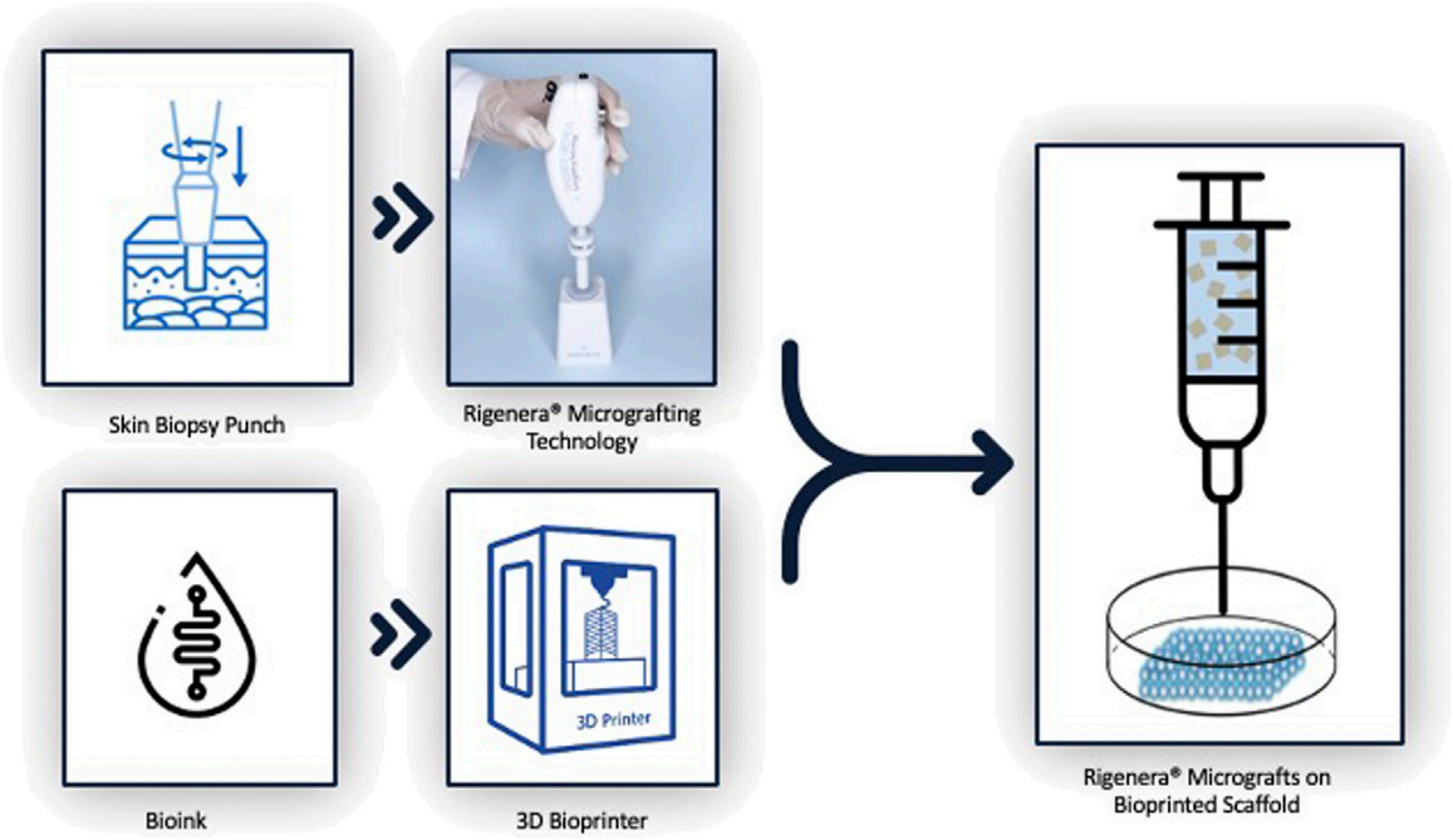
Process to obtain Rigenera ^®^ micrografts on the bioprinted scaffold.

Based on this research, it would be advantageous to fully exploit the 3D bioprinting technology together with the Rigenera^®^ technology with the aim of obtaining and engineering biological and biocompatible skin substitutes with an enhanced regeneration efficiency that could be applied to astronauts’ wounds during future interplanetary space missions, as shown in Figure 1.

In recent years, one of the major challenges has been the realization of a device that can be considered a novel effective countermeasure for severe skin injuries. The implementation of such a device could overcome the common treatment disadvantages, such as telemedicine that, despite its effective use on the ISS, it would have an increased communication delay when the astronauts get further from Earth, the high amount of tissue required, and the long sample preparation times (Farkas and Farkas, 2021; Niimi et al., 2022).

Obtaining an effective skin substitute that can be readily used, even in space missions, requires easy-to-use equipment and materials. Indeed, the characterization of a proper formula for an appropriate 3D skin substitute bioink is crucial. The term bioink refers to biomaterials that possess good printability characteristics and allow cells to survive when mixed with the bioink itself (Weng et al., 2021). Indeed biomaterials suitable for skin bioprinting, such as collagen, hyaluronic acid (HA), agarose, and alginate, possess good biocompatibility, promote cell adhesion, proliferation, and migration, do not cause inflammation, and can be enzymatically degraded. Moreover, they can be printed at low temperatures, significantly decreasing cellular stress (Murphy et al., 2013; Li et al., 2016; Weng et al., 2021).

**FIGURE 2:**
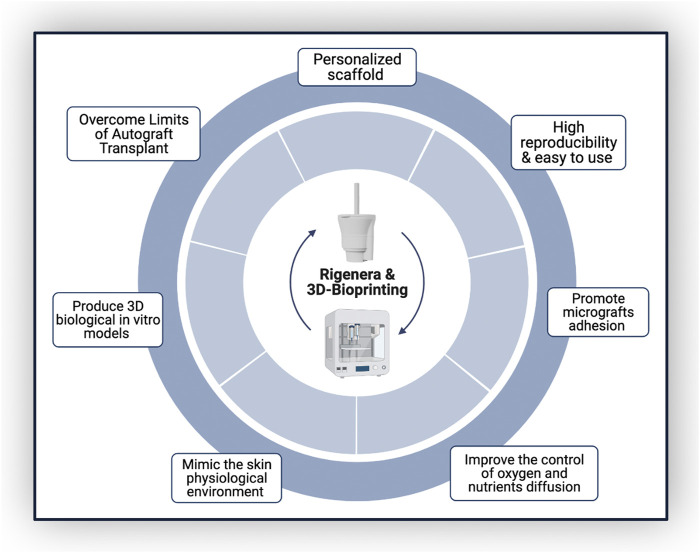
Applicability and suitability of the Rigenera^®^ technology with 3D bioprinting.

Combining Rigenera^®^ technology and 3D bioprinting would take advantage of both their high reproducibility and ease of use, allowing astronauts to produce personalized scaffolds in space, avoiding prior skin substitute production on Earth. This approach would allow mechanical scaffold features to be modulated to mimic biological tissues and, parallelly, improve the control of oxygen and nutrient diffusion, as well as promote cell adhesion and proliferation (Figure 2). In fact, AMGs could be applied directly over the wound, enhancing healing time and preventing fibrotic scar formation (Giaccone et al., 2014; Baglioni et al., 2016).

The experimental part is divided into a three-step cascade: in the first two steps, the scaffold will be designed (engineered in Computer Aided Design—CAD) and then bioprinted, layer-by-layer, into a fibrin hydrogel, and finally, AMGs will be seeded on it. To analyze the efficiency of hereby proposed mixed technology, AMGs will be applied on scratch-damaged bioprinted constructs, under the presence or absence of µG. Furthermore, to mimic the condition whereby an injured astronaut would come back to Earth to receive healthcare, the scratch-damaged bioprinted bioconstruct will be manufactured in µG conditions and subsequently, AMGs will be applied in a standard atmosphere environment. The goal of the above-mentioned experimental setup is to analyze a possible change in cell migration capability, in the presence and absence of AMGs and µG. The aim of this prospective research is to perform studies that can demonstrate, during µG tests, any alterations in pathways involved in skin regeneration confirmed through gene expression and microRNA analysis (Banerjee et al., 2011; Balli et al., 2020; Cialdai et al., 2020). Hence, the novel bioconstruct hereby proposed could *in vitro* predict the skin-related alterations in astronauts during µG exposure and be used as a reference model for the in-space construction of personalized patches to ameliorate the regeneration of damaged tissues. Prior to performing an *in vivo* study, it is advisable to proceed with *ex vivo* experiments, for instance, Botchkareva et al. devised an *ex vivo* model for testing WH-promoting compounds. Similarly, an experimental setup could be adopted using the *ex vivo* tissue alone under µG conditions as the negative control, the *ex vivo* tissue under µG conditions treated with AMGs as the test group, and the *ex vivo* tissue treated with AMGs in Earth gravity conditions as the positive control (Botchkareva and Westgate Editors, 2022).

## 3 Discussion

Effective WH is a challenging task to be achieved under normal gravity, and it becomes even more complex in µG conditions. Wounds that could arise in incidents on routine spaceflights may potentially affect astronauts’ health. Based on medical data collected in past spaceflights, skin alterations cause significant trauma to crew members (Singer and Boyce, 2017). It, therefore, becomes crucial to better understand how to minimize and treat skin deterioration in spaceflight (Riwaldt et al., 2021).

Currently, many different techniques are being used to treat WH, resulting in different outcomes. Certainly, the split-thickness autologous skin grafting treatment is deemed to be the gold standard for full-thickness wounds, although it exhibits disadvantages such as the need for a secondary surgical site, limited availability, and low cost-effectiveness.

For the xenograft technique, where a graft from an animal is collected, the main limitations concern graft rejection and the risk of transmitting viruses or zoonotic disease (Oryan et al., 2017).

It is, therefore, necessary to identify an easy, quick, and clinical-effective technique to improve WH outcomes. Furthermore, considering a µG environment, such as the one in the ISS, or during a long-term space exploration mission, it is essential to find an application that is easy to learn by the staff present in order to minimize infections, human errors, and rejection. It is proposed that the Rigenera^®^ AMG technology, described here, could address these limitations. Therefore, with the aim to investigate the cellular and molecular mechanisms behind AMG technology under µG conditions, as a first approach, an *in vitro* WH model (fibroblasts scratch assay) could be analyzed, as Balli et al. (2020) and Cialdai et al. (2020) analyzed (Monici et al., 2011). Considering that both migrations of fibroblasts and extracellular matrix (ECM) production are essential to guarantee proper WH, it is interesting that fibroblast migration decreases in a µG, and ECM production is significantly altered (Monici et al., 2011). In this respect, we propose to investigate how AMGs could influence the above-mentioned WH model; in particular, taking into consideration that AMGs consist of progenitor cells, extracellular matrix, and growth factors, and that in normal g condition, a scratch assay with them was already performed by Balli et al. with awesome outcomes, a relation between the Rigenera^®^ technology and its capability to affect fibroblast migration under µG could be speculated and deserves to be further investigated (Trovato and Failla, 2016).

Once the macroscopic feasibility has been settled through a first-level assessment, a second-tier investigation should be followed. Several genes and microRNAs are involved in the process of WH, and evaluating their expression in conditions of normal gravity and µG should help the scientific community to better understand the biochemical reaction behind skin repair and WH. This could be more easily achieved by recreating 3D skin *in vitro* models. The development of a three-dimensional construct would mimic a suitable microenvironment so that each type of cell (associated with the scaffold and with the micrografts) can express its own phenotype and perform its own functions (Yannas, 2018; Negut et al., 2020; Pavez Loriè et al., 2021; Botchkareva and Westgate Editors, 2022). Furthermore, the bioprinting technology provides the design and the manufacturing of bioconstructs that are personalized based on the shape and size of the astronauts’ wounds and are biocompatible, biodegradable, and induce angiogenesis (Yu et al., 2019).

Nevertheless, considering the difficulty of being able to perform *in vivo* studies under µG conditions, an *ex vivo* experiment could help in collecting a larger amount of evidence. Data on changes in inflammation, tissue formation, and tissue remodeling would be better observed through an *ex vivo* study, creating the possibility to confirm the preliminary data from the *in vitro* tests.

It is also interesting to be able to exploit what is already present on the International Space Station (ISS) so as to consider not only the parameters such as the difference in gravity but also the presence of different radiations, oxygen levels, and bacteria in the microenvironment that is created inside the station. In fact, astronauts are exposed to different types of radiation, namely, the ionizing radiation (IR) particles outside the Earth’s magnetic field, particles derived by solar flares, and galactic cosmic rays. All these factors can influence the outcome of healing of a wound, and considering them would bring all the experiments as close as possible to the environment that a human being affected by a skin injury could actually find onboard the ISS (Pavez Loriè et al., 2021).

For these reasons, our future research will be focused on the development of *in vitro* and *ex vivo* experimental studies that can confirm the expected results depicted in this article and that can provide solid results that afford to perform further studies which can overcome the presented limitations.

## 4 Conclusion

In this perspective study, a deepened analysis of the current situation of WH in conditions of µG was performed. Considering the strong desire of humans to push the boundaries and reach other planets and the necessity of finding new resources outside the Earth’s space, it is now necessary to understand how to prevent wound degeneration and re-establish a balanced physiological condition even in the absence of injuries. The scientific community is only at the beginning of this long path, and many facets of this research deserve to be addressed.

## Data Availability

The raw data supporting the conclusion of this article will be made available by the authors, without undue reservation.
